# Caffeinated Gum Enhances External Work During Perceptually Regulated Cycling in Well-Trained Male Athletes

**DOI:** 10.3390/nu18213639

**Published:** 2026-10-31

**Authors:** Nan Chen, Yifei Xia, Linyin Xu, Jue Liu, Li Ding, Jin Huang, Yinhang Cao, Yixiang Peng

**Affiliations:** 1 Department of Rehabilitation, The Second Affiliated Hospital, Zhejiang University School of Medicine, Hangzhou 310030, China; 2523139@zju.edu.cn (N.C.); 2523084@zju.edu.cn (Y.X.); 2 Faculty for Physical Education, Shanghai International Study University, Shanghai 201620, China; 02637@shisu.edu.cn; 3 Department of Rehabilitation Medicine, Huashan Hospital, Fudan University, Shanghai 200437, China; susanjue@126.com; 4 School of Athletic Performance, Shanghai University of Sport, Shanghai 200438, China; lidingsus@163.com (L.D.); 2421852025@sus.edu.cn (J.H.); 5 Faculty of Health Sciences and Sports, Macao Polytechnic University, Macao, China

**Keywords:** caffeine, ergogenic aid, exercise metabolism, self-selected intensity, perceived exertion, cycling

## Abstract

Background: While caffeine may lower ratings of perceived exertion (RPE) or increase power output at a given RPE, whether caffeinated gum produces these effects during perceptually regulated exercise remains unclear. Aim: This study examined whether caffeinated gum increases self-selected cycling output at regulated-RPE in well-trained male athletes. Methods: Twelve well-trained male athletes (age: 21 ± 2 years; body mass: 77.3 ± 6.0 kg; VO_2peak_: 47 ± 4 mL/kg/min) completed randomized, double-blind, placebo-controlled crossover trials separated by 7 days. Athletes chewed caffeinated gum (6 mg/kg) or placebo for 15 min before two 10 min cycling stages at RPE 4 and 7 (0–10 OMNI Scale). Cadence, power output, oxygen uptake, heart rate, and blood lactate concentration were measured. Results: Caffeinated gum significantly increased cadence (RPE 4: 72 ± 7 vs. 66 ± 10 rpm, *p* = 0.02; RPE 7: 77 ± 9 vs. 71 ± 11 rpm, *p* = 0.04), total work (53 ± 10 vs. 48 ± 10 kJ, *p* < 0.01; 87 ± 12 vs. 77 ± 14 kJ, *p* < 0.01), mean power output (88 ± 16 vs. 79 ± 16 W, *p* < 0.01; 146 ± 20 vs. 129 ± 24 W, *p* < 0.01) and peak power output (97 ± 18 vs. 88 ± 17 W, *p* < 0.01; 166 ± 27 vs. 147 ± 29 W, *p* = 0.01). Oxygen uptake was also higher in the caffeine condition at both RPE 4 (1.58 ± 0.12 vs. 1.42 ± 0.16 L/min, *p* = 0.005) and RPE 7 (2.17 ± 0.25 vs. 2.01 ± 0.22 L/min, *p* = 0.045), whereas heart rate and blood lactate concentration did not differ significantly between conditions (*p* > 0.05). Conclusion: Caffeinated gum (6 mg/kg) enhances self-selected cycling work at prescribed RPEs, indicating that it alters perceptually regulated exercise intensity in well-trained males.

## 1. Introduction

Caffeine has gained increasing popularity among athletes as an ergogenic aid, owing to its convenience, rapid absorption, and well established performance benefits [1,2,3]. Its ergogenic effects are largely attributed to antagonism of central adenosine A_1_ and A_2_A receptors [4,5,6]. By blocking adenosine-mediated signaling, caffeine may modulate neuronal activity, arousal, and fatigue-related processes, potentially influencing exercise-related perceptions [7]. This receptor blockade may attenuate pain perception during exercise [8] and reduce ratings of perceived exertion (RPE) at a given workload [9]. Importantly, exercise-related pain and perceived effort are distinct perceptual constructs, and caffeine-induced changes in one should not necessarily be interpreted as equivalent changes in the other [10]. These effects may be particularly relevant in sport, as RPE is widely used by athletes and coaches to prescribe and monitor exercise intensity [11].

RPE represents an individual’s subjective perception of exercise intensity and is commonly assessed using instruments such as the Borg 6–20 scale and the OMNI 0–10 scale, the latter incorporating verbal and pictorial descriptors [11,12]. The physiological basis of perceived exertion remains debated, with different theoretical models emphasizing the contributions of central motor commands, sensory feedback, and cognitive processes to exercise-related perceptions [13]. In particular, signals associated with central motor command have been proposed to contribute to the conscious perception of effort, whereas peripheral sensory feedback and exercise-related discomfort may influence other aspects of the exercise experience [14]. Although RPE generally increases with mechanical workload and associated physiological strain, the relationship between perceived exertion and external mechanical output may vary across individuals and experimental conditions [15]. RPE-regulated exercise involves adjusting workload to maintain a prescribed RPE [16]. Unlike fixed-workload exercise, in which mechanical demand is externally prescribed and RPE is measured as a perceptual response, RPE-regulated exercise requires individuals to adjust their workload according to a predetermined perceptual target [17]. Given that caffeine may reduce perceived exertion during fixed-intensity exercise [18,19], individuals may perceive a given workload as less demanding following caffeine ingestion. Under such conditions, participants may increase their self-selected workload until the prescribed RPE is reached, potentially producing greater mechanical power at an equivalent perceived intensity. This increase in mechanical output may be accompanied by greater cardiorespiratory and metabolic demands, despite the maintenance of a similar reported RPE. Although previous studies have reported increased mechanical output during caffeine-supplemented RPE-regulated exercise, the physiological mechanisms underlying this response remain uncertain, particularly regarding the contributions of central motor drive and neuromuscular function. Therefore, findings from fixed-workload exercise cannot be directly generalized to RPE-regulated exercise, highlighting the need to examine caffeine-induced changes in self-selected mechanical output under prescribed perceptual conditions.

One study reported that caffeine ingestion (6 mg/kg) significantly increased self-selected cycling workloads by approximately 17% and 16% during 20 min bouts performed at OMNI RPE 4 and 7, respectively [20]. Conversely, Cole et al. [21] found no significant change in workloads using a similar caffeine dose during 30 min of RPE-regulated cycling, comprising 10 min each at RPE 9, 12, and 15 on the Borg 6–20 RPE scale. More recently, Mask et al. [22] examined the effects of caffeinated gum (4.8 ± 0.7 mg/kg) in recreationally active females with low habitual caffeine intake and observed no significant ergogenic benefit on self-selected cadence at RPE 4 and 7. These contrasting findings suggest that caffeine does not consistently increase self-selected exercise output during RPE-regulated cycling. However, differences in caffeine administration, participant characteristics, exercise protocols, RPE scales, and outcome measures complicate direct comparisons. Caffeine delivery and administration timing may influence its absorption profile, whereas differences in participant characteristics and exercise protocols may contribute to variability in perceptual and performance response [23]. Moreover, Borg 6–20 and OMNI 0–10 ratings are not directly interchangeable because of their different perceptual anchors [11,12]. Importantly, self-selected cadence does not necessarily reflect mechanical power output, which depends on both angular velocity and torque. Although these methodological differences may contribute to the inconsistent findings, their relative contributions remain uncertain. Clarifying caffeine-induced changes in self-selected workload and associated physiological responses is therefore important for interpreting and applying RPE-based exercise prescription.

The selection of RPE 4 and RPE 7 on the OMNI 0–10 scale was intended to examine caffeine-induced responses at two distinct perceptual intensities. Validation research has supported the use of the adult OMNI Cycle Scale to assess perceived exertion across different cycling intensities [12]. These targets represent comparatively lower and higher levels of perceived exertion, respectively, and are consistent with previous RPE-regulated cycling protocols [20,22]. Higher perceived intensity during cycling is generally associated with greater mechanical workload and cardiorespiratory demands, including increased oxygen uptake, heart rate, and pulmonary ventilation [11,12]. However, the physiological responses associated with a given RPE vary with individual fitness, exercise experience, and perceptual calibration; thus, RPE 4 and RPE 7 cannot be assumed to correspond to fixed percentages of maximal oxygen uptake or specific metabolic thresholds. The use of these two perceptually separated targets was intended to examine whether caffeine-related changes in self-selected workload and physiological responses were evident under different levels of perceived exercise demand.

Caffeinated chewing gum provides an alternative caffeine delivery strategy that may facilitate relatively rapid absorption through the oral mucosa, although caffeine availability depends on dose and administration timing [24]. Despite previous investigations of caffeine during RPE-regulated cycling, evidence concerning the effects of caffeinated gum on objectively measured mechanical output and associated physiological responses in well-trained male athletes remains limited. In particular, it is unclear whether caffeinated gum alters the relationship between prescribed perceived exertion and self-selected cycling power, and whether any resulting increase in mechanical output is accompanied by greater cardiorespiratory demands. Therefore, this study examined whether caffeinated gum ingestion alters self-selected power output and associated physiological responses in well-trained males during cycling at RPE 4 and 7. We hypothesized that, compared with placebo, caffeinated gum would increase self-selected cycling power, including peak power output (PPO) and mean power output (MPO), at both RPE 4 and RPE 7, accompanied by greater cardiorespiratory responses consistent with the higher external workload. No directional hypothesis was proposed regarding whether the magnitude of these effects would differ between RPE 4 and RPE 7.

## 2. Methods

### 2.1. Participants

An a *priori* power analysis was conducted using G*Power (version 3.1.9.7), with cadence specified as the primary outcome because participants regulated exercise intensity by adjusting cadence against a fixed resistance, making cadence the directly controlled variable underlying changes in mechanical output. An F-test for repeated-measures ANOVA (within factors; one group and four repeated measurements) was specified with an assumed effect size of f = 0.51, calculated from the cadence data reported by Cao et al. [25], α = 0.05, power (1 − β) = 0.80, a correlation among repeated measures of 0.50, and no sphericity correction of ε = 1.0. This analysis indicated a minimum required sample size of seven participants (actual power = 0.83). However, the sample size calculation was based on a one-factor repeated-measures ANOVA, whereas the final analyses employed a two-factor repeated-measures ANOVA to examine condition and RPE stage effects separately. Therefore, the original sample size estimation may not fully reflect the statistical power of the factorial analyses. Twelve well-trained male athletes were recruited (age: 21.1 ± 2.4 years; body mass: 77.3 ± 6.0 kg; height: 182.7 ± 3.6 cm; VO_2peak_: 46.8 ± 4.4 mL/kg/min; and training volume: 6.8 ± 3.3 time/week). According to the participant classification framework proposed by Mckay et al. [26], all participants were categorized as Tier 3 athletes (National Level). The sample comprised basketball (*n* = 8), soccer (*n* = 2), and rugby (*n* = 2) athletes, all of whom engaged in structured, periodized training and regular competition and had competed at the national level. Although their VO_2peak_ values were relatively modest compared with those typically reported in endurance-trained athletes, their classification was based on competitive level and training background rather than aerobic capacity alone. Given the intermittent nature of their respective sports, VO_2peak_ may not fully reflect their overall training status. Participants were non-smokers, were not taking prescription medications, and had not engaged in altitude training during the preceding 6 months. Habitual caffeine intake was assessed using the Food Frequency Questionnaire and a caffeine intake habit classification table [27,28]. All participants were classified as low habitual caffeine consumers (<25 mg/day). The study was approved by the Ethics Committee of Shanghai University of Sport (No. 102772025RT200) and conducted in accordance with the Declaration of Helsinki. The study was retrospectively registered with the ISRCTN registry (ISRCTN13801569; https://doi.org/10.1186/ISRCTN13801569). Retrospective registration was undertaken because the study was initially considered an exercise physiology investigation rather than a clinical trial requiring prospective registration. Following clarification of the ICMJE clinical trial registration requirements, the study was registered retrospectively to ensure transparency and compliance with journal requirements. All participants provided verbal and written informed consent.

### 2.2. Study Design

A double-blind, counterbalanced, randomized crossover design was employed to examine the effects of commercially available caffeinated chewing gum on self-selected cycling performance and physiological responses during RPE-regulated exercise. Participants reported to the laboratory on five separate occasions, comprising a preliminary session for VO_2peak_ assessment, two familiarization sessions, and two main experimental trials. The order of the caffeine and placebo trials was randomized and counterbalanced. Experimental trials were scheduled at the same time of day for each participant to minimize the impact of circadian variation, and were separated by 7 days to allow adequate recovery and prevent carryover effects [29]. Participants were instructed to abstain from caffeine and alcohol and avoid strenuous exercise for 24 h before each trial while maintaining their usual diet and replicating their dietary intake across trials. Participants reported to the laboratory well rested and in a non-fasted state, having refrained from strenuous physical activity for ≥24 h. Compliance with these pre-test instructions was verified verbally before each visit.

**Figure 1:**
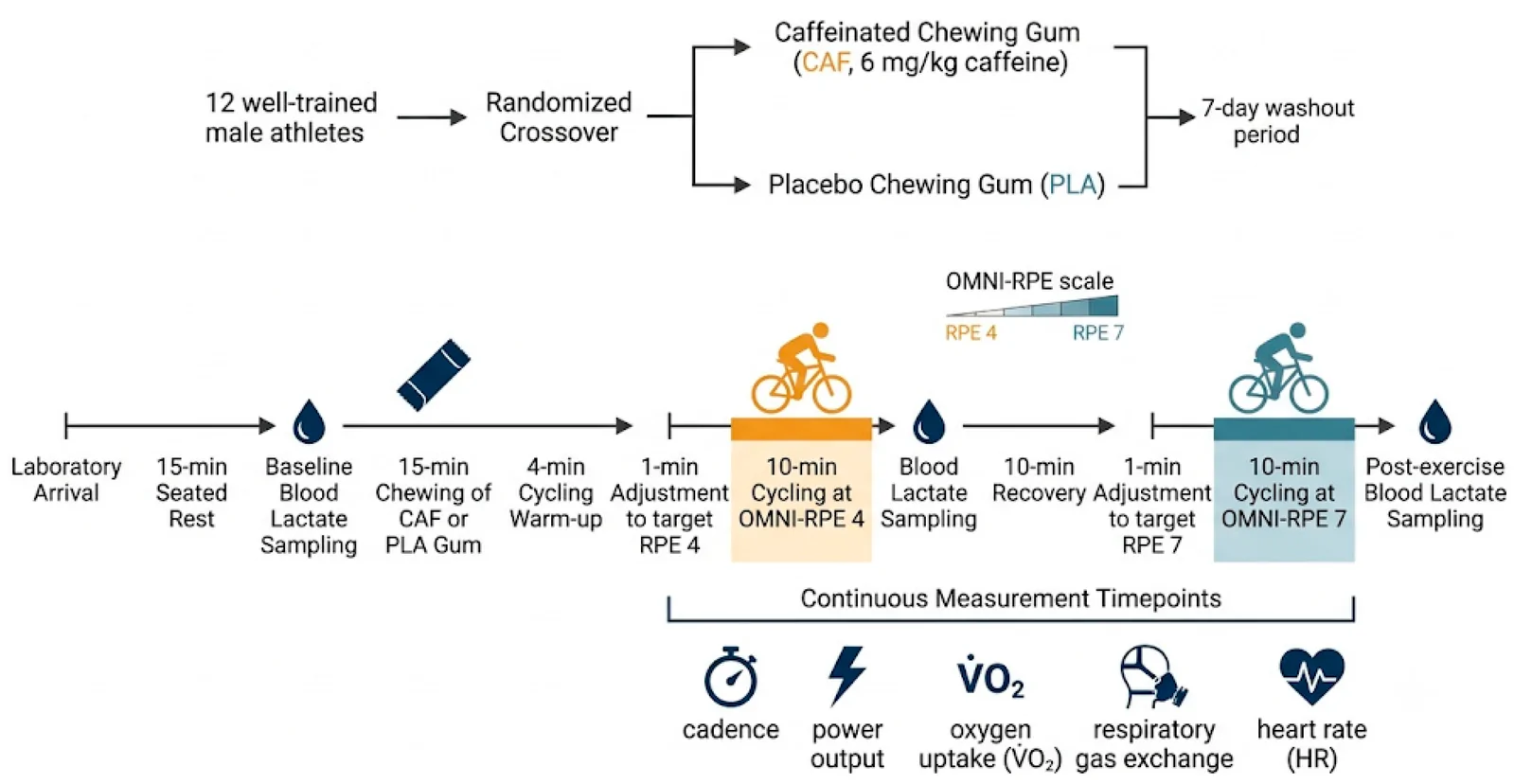
Study design. Twelve well-trained male athletes completed randomized crossover trials with caffeinated gum (CAF; 6 mg/kg) or placebo (PLA), separated by a 7-day washout. Each trial comprised 10 min of cycling at RPE 4, 10 min of recovery, and 10 min at RPE 7. Cadence, power output, VO_2_, respiratory gas exchange, and HR were monitored continuously, while blood lactate concentration was assessed at baseline and after each RPE stage.

Upon laboratory arrival, participants rested in a seated position for 15 min before a baseline capillary blood sample was collected from a pre-warmed fingertip for BLC analysis. They then chewed either caffeinated or placebo gum for 15 min. Once instrumented with a metabolic cart (Metalyzer 3B; Cortex, Leipzig, Germany) and HR monitor (Vantage NV; Polar, Kempele, Finland), participants performed a 4 min warm-up at the same fixed resistance as the RPE 4 trial, followed by a 1 min adjustment period to establish the workload corresponding to RPE 4. Participants then completed 10 min of cycling at RPE 4, followed by collection of a second blood sample. After a 10 min recovery period, participants completed a further 1 min adjustment period before cycling for 10 min at RPE 7. The RPE 4 stage always preceded the RPE 7 stage (Figure 1).

### 2.3. Supplementation Protocol

Upon baseline blood sampling, athletes chewed either caffeinated gum or an appearance-matched placebo gum for 15 min. The caffeine treatment (Military Energy Gum; MarketRight Inc., Plano, TX, USA) provided 6 mg/kg body mass, with each stick containing 100 mg of caffeine. This dose was selected based on previous research demonstrating ergogenic effects at 6 mg/kg [25]. The individualized caffeine dose was calculated according to each participant’s body mass. Participants received the required number of whole gum sticks, and any remaining fractional dose was obtained by weighing and portioning an additional stick using an analytical balance (Sartorius, Göttingen, Germany). The caffeine content of fractional portions was estimated assuming a uniform distribution of caffeine within each stick. To maintain double-blinding, the placebo treatment (Eclipse; Wm. Wrigley Jr. Company, Chicago, IL, USA) was matched to the caffeinated gum in appearance, color, texture, and flavor. Placebo gum was similarly weighed and portioned to match gum quantity and mass between conditions. After chewing the gum for 15 min to facilitate buccal caffeine absorption, participants discarded it before commencing the exercise protocol.

### 2.4. Graded Test

VO_2peak_ was assessed using a graded exercise test. Following 3 min of seated rest, participants completed a 3 min warm-up at 50 W and rested for a further 2 min. The test commenced at 60 W, with workload increasing by 30 W/min until volitional exhaustion. Participants were instructed to maintain a cadence of 70 rev/min throughout the test. The test was terminated when cadence fell below 60 rev/min for >3 s despite strong verbal encouragement [30]. Peak oxygen uptake was defined as the highest 3 s mean recorded during the test. Saddle height on a Monark 828E cycle ergometer (Monark Exercise AB, Vansbro, Sweden) was adjusted to allow a slight knee flexion at the bottom dead center (6 o’clock position), while handlebar position was adjusted according to participant preference.

### 2.5. Mechanical Output Measures

Initial workloads were estimated using the ACSM metabolic equation for leg cycling [31]. Because pilot testing indicated that these estimates were not consistently sustainable during the 10 min RPE-production bouts, resistance was individualized during familiarization. Participants completed separate 5 min titration periods at RPE 4 and RPE 7, during which resistance was progressively increased or decreased according to reported RPE until the target RPE could be maintained. The resulting resistance at each RPE was then fixed and replicated across the caffeine and placebo trials. During the experimental trials, resistance remained fixed, while participants self-adjusted cadence to maintain the prescribed RPE. Cadence and power output were recorded throughout each 10 min cycling bout and averaged over 3 s intervals. Mean cadence and MPO were calculated by averaging all 3 s values across the entire bout, whereas PPO was defined as the highest 3 s averaged power value observed during the 10 min bout. Total work was calculated as the time integral of power output over the 10 min bout.

### 2.6. Ratings of Perceived Exertion

RPE was assessed using the 0–10 OMNI scale [11], which remained visible throughout each experimental trial. RPE 4 and RPE 7 were selected to represent two distinct levels of perceived exercise intensity, consistent with previous RPE-regulated cycling protocols [20,22]. These targets were used to examine caffeine-induced changes in self-selected mechanical output and associated physiological responses under comparatively lower and higher perceptual demands, without assuming fixed physiological intensity equivalents. During each 10 min cycling bout, participants reported their RPE at 1 min intervals and adjusted cadence as necessary to maintain the prescribed target (RPE 4 or 7). Cadence, power output, elapsed time, and other performance feedback were concealed from participants throughout each bout. For each participant, the mean RPE was calculated from the 10 ratings recorded during each exercise stage. Group means and standard deviations were then calculated from the 12 participant-level mean values.

### 2.7. Physiological Responses

Respiratory and gas-exchange variables, including oxygen uptake (VO_2_), carbon dioxide output (VCO_2_), respiratory exchange ratio (RER), minute ventilation (VE), tidal volume (TV), breathing frequency (BF), end-tidal O_2_ and CO_2_ pressures, and ventilatory equivalents for O_2_ and CO_2_, were measured breath-by-breath throughout each 10 min exercise stage using a metabolic cart (Metalyzer 3B; Cortex, Leipzig, Germany). Before each trial, the system was calibrated using a precision gas mixture (15% O_2_ and 5% CO_2_), and the flow meter was calibrated using a 3 L syringe. For the time-course analysis, single values corresponding to the prespecified timepoints at 2, 4, 6, 8, and 10 min were extracted from the continuous breath-by-breath recordings, with no temporal averaging around these timepoints. Separately, data collected across the entire 10 min stage were averaged to characterize the overall physiological response under each condition. No manual or predefined automated artifact-detection or removal procedures were applied before data extraction and statistical analysis. HR was recorded every 5 s using a HR monitor (Vantage NV; Polar, Kempele, Finland). Duplicate capillary blood samples were collected from a pre-warmed fingertip immediately before exercise, immediately after the RPE 4 stage, and immediately after the RPE 7 stage. BLC was determined using a Biosen C-Line analyser (EKF Diagnostics, Cardiff, UK), with the mean of the duplicate measurements used for analysis.

### 2.8. Statistical Analyses

Data are presented as mean ± standard deviation (SD). Normality was assessed using the Shapiro–Wilk test. For within-participant factors with more than two levels, sphericity was assessed using Mauchly’s test, and the Greenhouse–Geisser correction was applied when the assumption of sphericity was violated. For mechanical and cardiorespiratory outcomes averaged across each 10 min exercise stage, two-way repeated-measures ANOVA was used to examine the main effects of condition (caffeine vs. placebo) and RPE stage (RPE 4 vs. RPE 7), as well as the condition × RPE stage interaction. For the time-course physiological data, separate two-way repeated-measures ANOVAs were conducted for the RPE 4 and RPE 7 stages, with condition (caffeine vs. placebo) and time (2, 4, 6, 8, and 10 min) as within-participant factors. The main effects of condition and time, as well as the condition × time interaction, were examined. When a significant condition × time interaction was detected, pairwise comparisons between conditions at individual timepoints were performed with Bonferroni adjustment for multiple comparisons. Where no significant condition × time interaction was present, the main effect of condition is reported. BLC was analyzed using a separate two-way repeated-measures ANOVA with condition (caffeine vs. placebo) and time (baseline, post-RPE 4, and post-RPE 7) as within-participant factors. Significant condition × time interactions were followed by Bonferroni-adjusted pairwise comparisons. Effect sizes were estimated using partial eta squared (ηp^2^). Statistical significance was set at *p* < 0.05. All statistical analyses were performed using IBM SPSS Statistics (Version 28.0; IBM Corp., Armonk, NY, USA), and graphical visualizations were generated using R software (version 4.2.0; R Foundation for Statistical Computing, Vienna, Austria).

## 3. Results

### 3.1. Mechanical Output Variables

**Figure 2:**
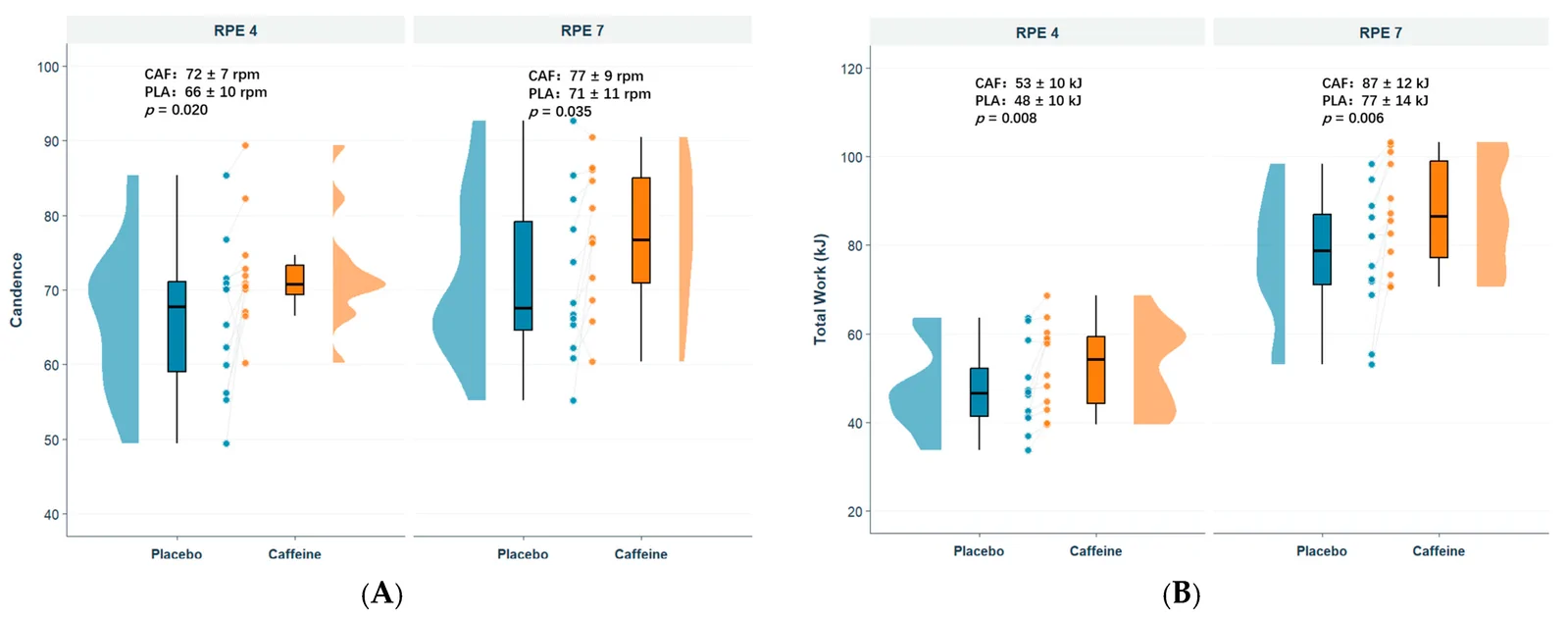

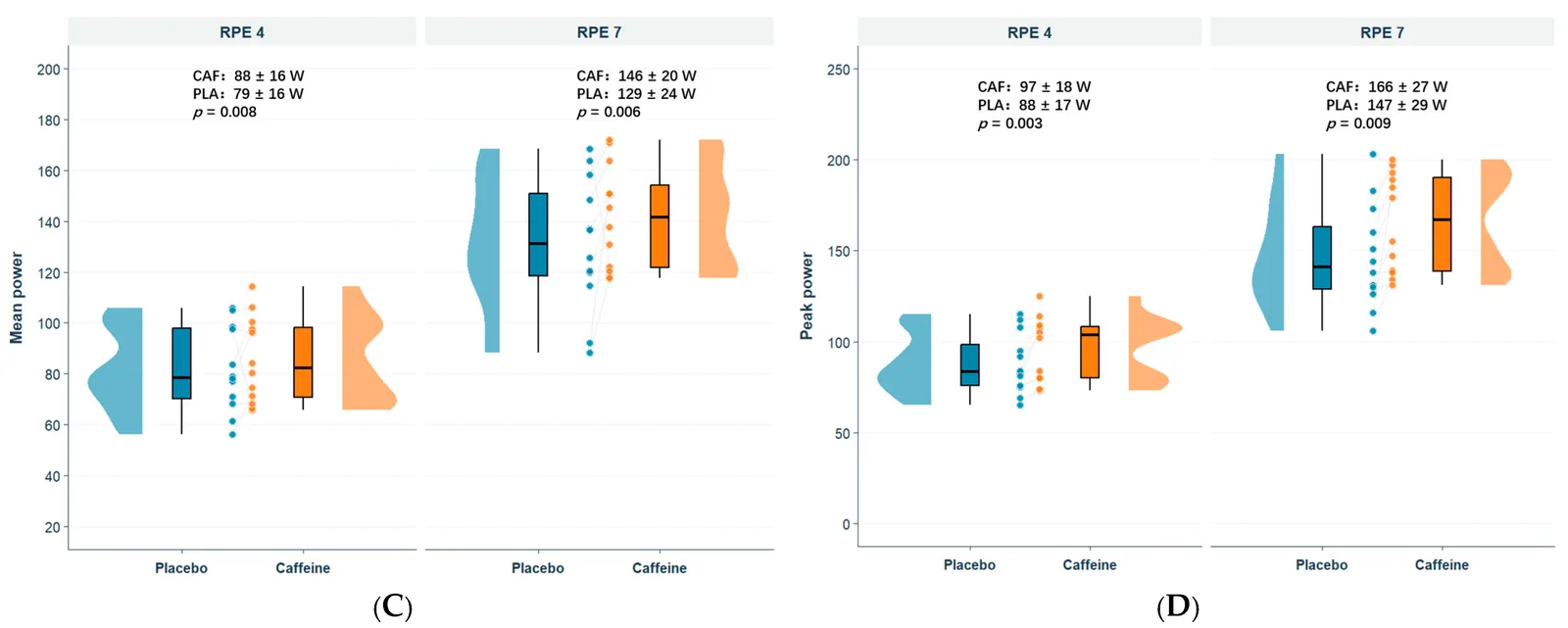
Mechanical outcomes at RPE 4 and RPE 7 during caffeine (CAF) and placebo (PLA) trials. Values are presented as mean ± SD. Panels show cadence (**A**), total work (**B**), mean power (**C**), and peak power (**D**). Raincloud plots display individual observations, boxplots, and distribution densities. Stage 1 and Stage 2 correspond to RPE 4 and RPE 7, respectively.

Cadence showed a significant main effect of condition (F = 8.598, *p* = 0.014, partial η^2^ = 0.439). Cadence was significantly higher in the caffeine than placebo condition at both RPE 4 (72 ± 7 vs. 66 ± 10 rpm; *p* = 0.020) and RPE 7 (77 ± 9 vs. 71 ± 11 rpm; *p* = 0.035) (Figure 2A). Furthermore, PPO was significantly higher with caffeine at both RPE 4 (97 ± 18 vs. 88 ± 17 W, *p* = 0.003) and RPE 7 (166 ± 27 vs. 147 ± 29 W, *p* = 0.009) (Figure 2D). Similarly, total work was greater with caffeine at RPE 4 (53 ± 10 vs. 48 ± 10 kJ, *p* = 0.008) and at RPE 7 (87 ± 12 vs. 77 ± 14 kJ, *p* = 0.006) (Figure 2B). Consistent with total work, MPO was significantly higher with caffeine at both RPE 4 (88 ± 16 vs. 79 ± 16 W; *p* = 0.008) and RPE 7 (146 ± 20 vs. 129 ± 24 W; *p* = 0.006) (Figure 2C)

### 3.2. Physiological Responses

**Figure 3:**
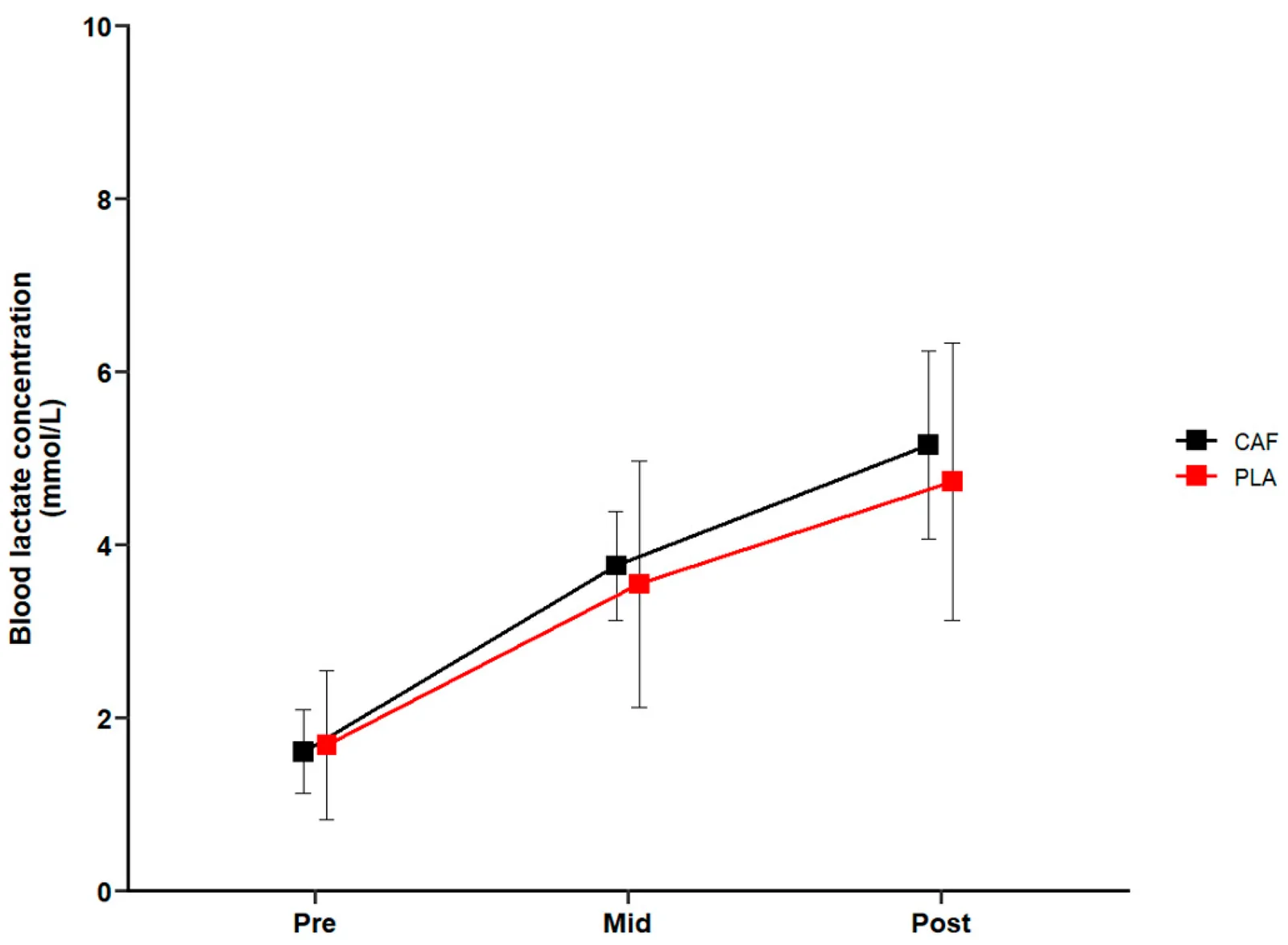
Blood lactate concentration across timepoints during caffeine (CAF) and placebo (PLA) trials. Values are presented as mean ± SD. Pre, pre-exercise; Mid, mid-exercise (immediately following RPE 4 stage); Post, post-exercise.

VO_2_ was significantly higher in the caffeine condition than in the placebo condition at both RPE 4 (1.58 ± 0.12 vs. 1.42 ± 0.16 L/min, *p* = 0.005) and RPE 7 (2.17 ± 0.25 vs. 2.01 ± 0.22 L/min, *p* = 0.045). Similarly, percentage of predicted normal VO_2_ was significantly higher in the caffeine condition than in the placebo condition across RPE 4 (F = 12.283, *p* = 0.005, partial η^2^ = 0.528) and RPE 7 (F = 4.934, *p* = 0.048, partial η^2^ = 0.310) (Table 1 and Table 2). In contrast, no significant differences in BLC were observed between conditions at either RPE 4 or RPE 7 (both *p* > 0.05) (Figure 3). HR did not differ significantly between the caffeine and placebo conditions at either RPE 4 or RPE 7 (both *p* > 0.05) (Table 1 and Table 2). At RPE 4, repeated-measures ANOVA demonstrated significant treatment effects for relative VO_2_ (F = 11.395, *p* = 0.006, partial η^2^ = 0.509), VE (F = 5.406, *p* = 0.040, partial η^2^ = 0.330), VCO_2_ (F = 8.123, *p* = 0.016, partial η^2^ = 0.425), and BF (F = 5.021, *p* = 0.047, partial η^2^ = 0.313). TV showed a trend toward significance (F = 4.841, *p* = 0.050, partial η^2^ = 0.306) (Table 1). At RPE 7, significant treatment effects were observed for ventilatory equivalent for CO_2_ (F = 6.113, *p* = 0.031, partial η^2^ = 0.357; Supplementary Table S3), whereas relative VO_2_ showed a trend toward significance (F = 4.823, *p* = 0.050, partial η^2^ = 0.305) (Table 2). Overall, VO_2_ was significantly higher in the caffeine condition than in the placebo condition (F = 9.053, *p* = 0.012, ηp^2^ = 0.451), with similar increases observed for relative VO_2_, TV, VCO_2_, and ventilatory equivalent for CO_2_ (all *p* < 0.05; Table 2). No significant condition × time interactions were observed for the physiological variables at either RPE stage (all *p* > 0.05; Supplementary Table S4).

### 3.3. Achieved Ratings of Perceived Exertion

Achieved RPE values were consistently maintained at the prescribed targets throughout both exercise stages. Specifically, RPE values were 4.0 ± 0.0 during the RPE 4 stage and 7.0 ± 0.0 during the RPE 7 stage under both caffeine and placebo conditions.

## 4. Discussion

This study is the first to assess the effects of caffeinated gum on exercise performance and physiological responses in well-trained male athletes during RPE-regulated exercise (RPE4 and RPE7). Main findings indicated that caffeinated gum significantly increased cadence, PPO, MPO, and total work. Additionally, VO_2_, TV, the percentage of predicted normal VO_2_, and VCO_2_ were also significantly elevated. Our results partially support our hypothesis, showing that caffeinated gum increased self-selected mechanical output and associated physiological responses compared with placebo across the prescribed RPE levels.

### 4.1. Mechanical Output

These findings differ from those of Mask et al. [22], who reported no effect of caffeinated gum on cadence at RPE 4 or 7 in recreationally active females. This discrepancy may reflect differences in participant characteristics, as their sample included individuals with varying habitual caffeine intake, whereas the present study involved well-trained males with low habitual caffeine intake. Training status, habitual caffeine exposure, and sex-related differences in caffeine pharmacokinetics may influence the ergogenic response to caffeine [32,33,34]. However, as these factors were not directly examined, they should be considered potential rather than definitive explanations for the contrasting findings.

Caffeinated gum increased self-selected mechanical output at both RPE 4 and RPE 7, with improvements of approximately 10–13% in PPO and MPO. The magnitude of these changes suggests that caffeine enabled participants to self-select a greater mechanical output at the same prescribed RPE. These findings are consistent with those of Langford et al. [20], who reported approximately 16–17% greater self-selected cycling workloads following caffeine ingestion during RPE-regulated exercise. Although the improvements observed in the present study were slightly smaller, their magnitude may still be practically relevant, as athletes generated greater mechanical output while maintaining the same prescribed RPE. However, the absence of protocol-specific reliability data limits our ability to determine whether these changes exceed normal measurement variability. Unlike fixed-workload exercise, RPE-regulated cycling allows participants to adjust mechanical output to maintain a prescribed perceptual target. If caffeine reduces perceived exertion at a given workload, participants may select higher power outputs to reach the same target RPE, potentially increasing physiological demands. The observed improvements may be partly explained by caffeine’s central effects. By antagonizing adenosine receptors [5,35], caffeine may modulate neuronal excitability and attenuate exercise-related discomfort, potentially allowing athletes to select greater mechanical output at the same RPE [4,8]. Although exercise-related discomfort and perceived effort are distinct constructs, signals associated with central motor command may contribute to the conscious perception of effort [36]. Thus, caffeine could theoretically influence the relationship between perceived effort and self-selected workload, although this mechanism was not directly assessed.

Caffeine may also influence neuromuscular function [9], potentially affecting muscle activation or force production; however, greater cycling power alone does not establish increased central motor drive or motor-unit recruitment. Similarly, Duncan et al. [37] reported greater subjective readiness to invest mental and physical effort following caffeine ingestion, suggesting that changes in motivational state may also contribute to increased self-selected workloads [15]. However, these mechanisms remain speculative because neural activation, motor drive, and exercise-related discomfort were not directly assessed in the present study.

### 4.2. Physiological Responses

HR was slightly higher with caffeine than placebo at both RPE 4 and RPE 7, although the differences were not statistically significant (Table 1 and Table 2). This finding is consistent with Langford et al. [20], who also observed no significant effect of 6 mg/kg caffeine on HR during RPE-regulated exercise. Similarly, Green et al. [38] reported HR differences approaching statistical significance following caffeine ingestion during RPE-regulated treadmill exercise. Robertson et al. [39] suggested that respiratory-related signals may contribute to perceived exertion, although their relative importance compared with cardiovascular signals remains uncertain. Nevertheless, the nonsignificant HR differences do not establish equivalent cardiovascular strain, and the contributions of cardiovascular and respiratory signals to RPE remain uncertain.

The higher VO_2_ and TV observed with caffeine should be interpreted in the context of the greater self-selected mechanical output. Higher cycling power generally requires greater metabolic energy turnover and oxygen demand, indicating that identical prescribed RPE targets do not necessarily correspond to equivalent physiological strain. Because workload was not matched between conditions, these responses may primarily reflect the increased metabolic demand associated with greater mechanical output rather than a direct physiological effect of caffeine. Similarly, the higher TV may reflect increased respiratory demand but does not necessarily indicate greater minute ventilation, which also depends on breathing frequency. Although caffeine has been reported to influence ventilatory control [40,41,42], the present design cannot distinguish its direct effects from those secondary to increased workload. Despite greater mechanical output following caffeine ingestion, BLC remained similar between conditions. This finding may reflect the complex balance between lactate production, transport, oxidation, and clearance, which collectively determine circulating lactate concentrations. Furthermore, the timing of blood sampling may have influenced the observed responses, as BLC may continue to change during the early post-exercise period. Therefore, the absence of significant differences in BLC should not be interpreted as evidence of improved metabolic efficiency or altered substrate utilization, as these mechanisms were not directly assessed. Differences between RPE 4 and RPE 7 should be interpreted cautiously, as the fixed stage order may have introduced effects of accumulated fatigue. Collectively, these findings suggest that caffeinated gum may alter the relationship between perceived exertion and external mechanical output, allowing greater power production and accompanying physiological demands at the same prescribed RPE.

### 4.3. Limitations and Additional Considerations

Several limitations should be acknowledged. First, the experimental design was restricted to acute, short-duration (10 min) bouts of submaximal cycling, with the 10 min duration selected to be consistent with the protocol used in previous studies [38,43]. Consequently, the findings cannot be extrapolated to exhaustive exercise performance (e.g., time-to-exhaustion trials) or the physiological adaptations associated with chronic caffeine ingestion over long-term training. Second, the exercise modality constrained self-regulated intensity: resistance was fixed at approximately 50% and 70% of VO_2peak_ for RPE 4 and RPE 7, respectively, while cadence was freely adjustable, allowing power output to vary between conditions. However, the inability to adjust resistance may limit the applicability of these findings to real-world pacing strategies. Additionally, because RPE 4 always preceded RPE 7, exercise intensity was confounded by stage order and potential accumulated fatigue. Thus, the two RPE stages should not be considered fully independent, and between-intensity comparisons should be interpreted with caution. Furthermore, protocol-specific test–retest reliability and minimal detectable change for mechanical output at OMNI RPE 4 and RPE 7 were not established. Although participants completed two familiarization sessions, the absence of reliability data limits our ability to determine whether the observed improvements exceeded typical measurement variability. Therefore, the practical significance of these changes should be interpreted cautiously. Additionally, the sample size calculation was based on a different repeated-measures ANOVA structure from that used in the final analyses, which may have affected the statistical power of the study. Third, the mechanistic understanding of metabolic responses is limited by the absence of direct blood plasma measurements for free fatty acids. Although the RER did not reveal a significant shift toward fat metabolism, the lack of direct free fatty acid quantification means that subtle alterations in substrate utilization cannot be definitively confirmed or ruled out. Additionally, the absence of artifact removal for breath-by-breath respiratory data may have increased the influence of occasional measurement artifacts on the time-course analyses. Fourth, although the study has now been retrospectively registered with the ISRCTN registry (ISRCTN13801569), the absence of prospective registration remains a methodological limitation. Finally, the participant cohort consisted exclusively of active males. Given potential sex-based differences in caffeine metabolism and physiological responses [44], these results cannot be broadly generalized to female populations, highlighting the need for future research to include more diverse cohorts. Future studies should directly assess neuromuscular activation, perceived discomfort, and metabolic responses under both RPE-regulated and workload-matched conditions to clarify the mechanisms underlying caffeine-induced changes in self-selected mechanical output.

### 4.4. Practical Implications

From a practical perspective, caffeinated gum may alter the relationship between perceived exertion and external workload during RPE-regulated exercise. In the present study, caffeine was associated with approximately 10–13% greater mechanical output at the same prescribed RPE, suggesting that athletes may perform more external work without a corresponding increase in perceived exertion. Consequently, coaches and practitioners should consider caffeine intake when prescribing, monitoring, or comparing RPE-based training sessions, as the same RPE may not reflect an equivalent external workload under caffeinated and non-caffeinated conditions. However, these findings should be interpreted cautiously due to the absence of protocol-specific reliability data, and their applicability is primarily limited to young, well-trained males with low habitual caffeine intake performing short-duration cycling following ingestion of caffeinated gum providing 6 mg/kg of caffeine.

## 5. Conclusions

The present study demonstrates that 6 mg/kg caffeinated gum increased self-selected mechanical output during RPE-regulated cycling in well-trained males, as reflected by greater cadence, PPO, MPO, and total work. These changes were accompanied by higher VO_2_ and TV, although these physiological differences may partly reflect the greater external workload rather than a direct effect of caffeine. Overall, caffeinated gum may alter the relationship between perceived exertion and external workload, allowing greater mechanical output to be achieved at the same prescribed RPE during short-duration, submaximal cycling.

## Figures and Tables

**Table 1:** Time course of physiological responses during cycling at RPE 4 on the 0–10 OMNI scale following caffeine gum or placebo.

Variables	Trial	Timepoint					Mean	*p*-Value
		2 min	4 min	6 min	8 min	10 min	Mean ± SD	Trial
VO_2_ (L/min)	CAF	1.62 ± 0.44	1.59 ± 0.30	1.60 ± 0.14	1.67 ± 0.22	1.55 ± 0.23	1.58 ± 0.12 *	0.005
	PLA	1.44 ± 0.13	1.47 ± 0.24	1.45 ± 0.32	1.51 ± 0.33	1.39 ± 0.13	1.42 ± 0.16	
VCO_2_ (L/min)	CAF	1.40 ± 0.43	1.39 ± 0.27	1.37 ± 0.12	1.45 ± 0.20	1.34 ± 0.20	1.37 ± 0.10 *	0.016
	PLA	1.23 ± 0.11	1.26 ± 0.23	1.28 ± 0.29	1.30 ± 0.30	1.19 ± 0.13	1.22 ± 0.17	
VE (L/min)	CAF	42 ± 11	44 ± 9	43 ± 5	45 ± 7	44 ± 8	42 ± 3 *	0.040
	PLA	37 ± 5	37 ± 8	40 ± 8	39 ± 8	37 ± 7	38 ± 6	
VT (L)	CAF	1.65 ± 0.39	1.60 ± 0.38	1.69 ± 0.43	1.66 ± 0.20	1.59 ± 0.30	1.65 ± 0.15 *	0.050
	PLA	1.47 ± 0.21	1.60 ± 0.25	1.81 ± 0.79	1.58 ± 0.30	1.40 ± 0.24	1.55 ± 0.15	
BF (breaths/min)	CAF	26 ± 5	28 ± 6	27 ± 6	27 ± 6	29 ± 9	27 ± 5 *	0.047
	PLA	26 ± 8	24 ± 6	25 ± 8	25 ± 5	28 ± 8	25 ± 5	
RER	CAF	0.86 ± 0.05	0.88 ± 0.03	0.87 ± 0.03	0.87 ± 0.03	0.87 ± 0.03	0.87 ± 0.02	0.586
	PLA	0.86 ± 0.05	0.86 ± 0.05	0.88 ± 0.04	0.86 ± 0.04	0.85 ± 0.03	0.86 ± 0.04	
VO_2_/kg (ml/kg/min)	CAF	21.1 ± 6.2	20.5 ± 3.7	20.6 ± 2.2	21.7 ± 3.8	20.2 ± 3.1	20.4 ± 2.1 *	0.006
	PLA	18.5 ± 1.7	19.0 ± 2.7	18.8 ± 4.0	19.5 ± 4.2	18.1 ± 1.9	18.3 ± 2.1	
HR (Beats/min)	CAF	117± 9	120 ± 8	123 ± 10	122 ± 10	124± 12	119 ± 9	0.072
	PLA	110 ± 15	114 ± 14	115 ± 15	115 ± 16	115 ± 16	113 ± 15	

Values are presented as mean ± SD. CAF, caffeine; PLA, placebo; RPE, rating of perceived exertion; VO_2_/kg, relative oxygen uptake; VE, minute ventilation; TV, tidal volume; RER, respiratory exchange ratio; VCO_2_, carbon dioxide elimination; BF, breathing frequency. *p*-values represent the main effect of condition; *, indicating statistical significance (*p* < 0.05).

**Table 2:** Time course of physiological responses during cycling at RPE 7 on the 0–10 OMNI scale following caffeine gum or placebo.

Variables	Trial	Timepoint					Mean	*P*-Value	
		2 min	4 min	6 min	8 min	10 min	Mean ± SD	RPE 7	Overall
VO_2_ (L/min)	CAF	2.23 ± 0.36	2.17 ± 0.34	2.33 ± 0.26	2.31 ± 0.54	2.20 ± 0.36	2.17 ± 0.25 *	0.045	0.012
	PLA	2.01 ± 0.40	1.96 ± 0.38	2.11 ± 0.34	2.05 ± 0.40	2.09 ± 0.27	2.01 ± 0.22		
VCO_2_ (L/min)	CAF	2.09 ± 0.39	1.99 ± 0.33	2.06 ± 0.30	2.02 ± 0.51	1.95 ± 0.34	1.93 ± 0.26	0.097	0.034
	PLA	1.82 ± 0.37	1.78 ± 0.32	1.87 ± 0.34	1.83 ± 0.39	1.86 ± 0.30	1.78 ± 0.25		
VE (L/min)	CAF	65 ± 15	66 ± 14	69 ± 12	69 ± 22	68 ± 18	62 ± 10	0.117	0.062
	PLA	54 ± 12	55 ± 13	60 ± 17	59 ± 15	61 ± 16	57 ± 11		
VT (L)	CAF	2.10 ± 0.50	2.15 ± 0.50	1.98 ± 0.28	1.87 ± 0.28	1.97 ± 0.37	1.99 ± 0.30	0.074	0.025
	PLA	1.94 ± 0.46	1.82 ± 0.42	1.93 ± 0.40	1.92 ± 0.45	1.85 ± 0.44	1.86 ± 0.26		
BF (breaths/min)	CAF	32 ± 7	33 ± 13	35 ± 6	37 ± 9	36 ± 12	34 ± 8	0.206	0.087
	PLA	29 ± 8	32 ± 9	33 ± 13	32 ± 11	35 ± 12	31 ± 7		
RER	CAF	0.92 ± 0.04	0.92 ± 0.03	0.88 ± 0.04	0.87 ± 0.03	0.89 ± 0.03	0.88 ± 0.03	0.917	0.771
	PLA	0.91 ± 0.06	0.90 ± 0.05	0.88 ± 0.05	0.89 ± 0.04	0.88 ± 0.04	0.88 ± 0.04		
VO_2_/kg (ml/kg/min)	CAF	29.0 ± 5.3	28.2 ± 5.9	30.4 ± 5.0	30.3 ± 8.7	28.6 ± 5.9	28.2 ± 4.1 *	0.050	0.012
	PLA	26.2 ± 5.5	25.2 ± 4.6	27.3 ± 5.1	26.8 ± 6.2	27.3 ± 4.5	26.2 ± 3.8		
HR	CAF	140 ± 14	146± 15	150 ± 15	151 ± 16	153 ± 17	140 ± 24	0.809	0.410
	PLA	134 ± 17	137 ± 19	141 ± 21	144 ± 21	145 ± 23	138 ± 19		

Values are presented as mean ± SD. CAF, caffeine; PLA, placebo; RPE, rating of perceived exertion; VO_2_/kg, relative oxygen uptake; VE, minute ventilation; TV, tidal volume; RER, respiratory exchange ratio; VCO_2_, carbon dioxide elimination; BF, breathing frequency. “RPE 7” *p*-values indicate the condition effect (CAF vs. PLA) at RPE 7, whereas “Overall” *p*-values indicate the condition effect across RPE 4 and RPE 7; *, indicating statistical significance (*p* < 0.05).

## Data Availability

The data supporting the findings of this study are available within the article and its Supplementary Materials.

## Supplementary Materials

The following supporting information can be downloaded at https://www.mdpi.com/article/10.3390/nu18213639/s1. Table S1: Mechanical Performance Outcomes; Table S2: Physiological Responses at RPE 4; Table S3: Physiological Responses at RPE 7; Table S4: Physiological Response Interaction Effects.
