# Supplementary material for: Caffeinated Gum Enhances External Work During Perceptually Regulated Cycling in Well-Trained Male Athletes
Source: Nutrients. 2026 Oct 31;18(21):3639. doi: 10.3390/nu18213639 (PMC13764918; doi:10.3390/nu18213639)
Supplement: Supplementary file 1 [file nutrients-18-03639-s001.zip › nutrients-4601234-supplementary.pdf]

Supplementary Material Table S1

Mechanical Performance Outcomes at RPE 4 and RPE 7 Following Caffeinated Gum or Placebo Ingestion.

Variables	RPE	Mean ± SD		*p*-value
		CAF	PLA	Trial
Candence (rpm)	4	72 ± 7	66 ± 10	0.020
	7	77 ± 9	71 ± 11	0.035
Total work (kJ)	4	53 ± 10	48 ± 10	0.008
	7	87 ± 12	77 ± 14	0.006
Mean power output (W)	4	88 ± 16	79 ± 16	0.008
	7	146 ± 20	129 ± 24	0.006
Peak power output (W)	4	97 ± 18	88 ± 17	0.003
	7	166 ± 27	147 ± 29	0.009

Supplementary Material Table S2

Time course of physiological responses during cycling at RPE 4 on the 0-10 OMNI scale following caffeine gum or placebo.

Variables	Trial	Timepoint					Mean	*p*-value
		2 min	4 min	6 min	8 min	10 min	Mean ± SD	Trial
VE/VO_2	CAF	23.5 ± 2.1	24.8 ± 2.8	24.6 ± 2.4	24.5 ± 2.6	25.6 ± 3.6	24.7 ± 2.0	0.171
	PLA	23.4 ± 2.9	22.5 ± 2.6	25.2 ± 2.9	23.5 ± 2.4	24.1 ± 3.8	23.6 ± 2.6	
VE/VCO_2	CAF	27.4 ± 2.0	28.3 ± 3.2	28.5 ± 2.7	28.3 ± 2.6	29.4 ± 3.8	28.2 ± 2.3	0.097
	PLA	27.2 ± 2.6	26.3 ± 2.6	28.6 ± 3.3	27.5 ± 2.7	28.2 ± 3.9	27.4 ± 2.7	
$PETO_2$ (mmHg)	CAF	103.8 ± 3.3	106.7 ± 3.3	105.8 ± 3.3	105.9 ± 3.9	106.8 ± 4.9	105.6 ± 2.8	0.231
	PLA	103.7 ± 3.9	102.7 ± 4.3	106.3 ± 3.7	104.3 ± 3.2	104.6 ± 4.8	104.2 ± 3.8	
$PETCO_2$ (mmHg)	CAF	39.7 ± 2.3	38.1 ± 2.8	38.6 ± 3.0	38.2 ± 2.6	37.2 ± 3.7	38.5 ± 2.4	0.159
	PLA	39.8 ± 2.9	40.7 ± 2.8	38.5 ± 3.1	39.3 ± 2.1	39.0 ± 3.3	39.4 ± 2.6	
$\%VO_{2norm}$ (%)	CAF	46.8 ± 13.6	46.2 ± 9.0	46.4 ± 4.3	48.3 ± 6.8	44.8 ± 6.6	45.7 ± 4.0*	0.005
	PLA	41.8 ± 4.0	42.7 ± 6.6	41.9 ± 9.8	43.8 ± 10.0	40.3 ± 3.9	41.1 ± 4.7	
VO_2/HR (ml/beat)	CAF	13.8 ± 3.9	13.3 ± 2.9	13.1 ± 1.9	13.8 ± 1.8	12.7 ± 2.3	13.3 ± 1.3	0.137
	PLA	13.3 ± 2.3	13.0 ± 2.7	12.6 ± 2.8	13.3 ± 2.8	12.3 ± 2.1	12.7 ± 1.5	

Values are presented as mean ± SD. CAF, caffeine; PLA, placebo; RPE, rating of perceived exertion; VO_2/HR, oxygen pulse; $\%VO_{2norm}$, percentage of predicted normal oxygen uptake; VE/VO_2, ventilatory equivalent for oxygen; VE/VCO_2, ventilatory equivalent for carbon dioxide. *p*-values represent the main effect of condition.

Supplementary Material Table S3

Time course of physiological responses during cycling at RPE 7 on the 0-10 OMNI scale following caffeine gum or placebo.

Variables	Trial	Timepoint					Mean	*p*-value	
		2 min	4 min	6 min	8 min	10 min	Mean ± SD	RPE 7	Overall
VE/VO_2	CAF	26.9 ± 3.0	28.1 ± 4.2	27.1 ± 3.0	27.2 ± 3.9	28.4 ± 5.1	26.6 ± 3.0	0.064	0.072
	PLA	25.0 ± 3.8	25.9 ± 5.0	25.9 ± 4.3	26.3 ± 3.8	26.5 ± 4.7	25.3 ± 2.7		
VE/VCO_2	CAF	29.3 ± 3.4	30.6 ± 5.0	30.8 ± 3.4	31.1 ± 4.4	32.1 ± 5.4	29.9 ± 3.3*	0.031	0.013
	PLA	27.6 ± 3.8	28.7 ± 4.1	29.4 ± 3.8	29.5 ± 3.0	29.9 ± 4.0	28.6 ± 2.5		
$PETO_2$ (mmHg)	CAF	108.8 ± 4.0	110.0 ± 5.2	109.1 ± 4.6	108.2 ± 5.4	109.7 ± 5.7	108.3 ± 3.5	0.094	0.132
	PLA	105.5 ± 5.7	106.8 ± 6.5	107.2 ± 5.8	107.4 ± 5.2	107.8 ± 6.4	106.5 ± 4.1		
$PETCO_2$ (mmHg)	CAF	37.8 ± 3.7	36.7 ± 4.6	36.2 ± 3.7	36.6 ± 3.9	35.7 ± 4.7	36.9 ± 3.0	0.055	0.076
	PLA	39.8 ± 3.7	38.7 ± 4.4	37.7 ± 4.1	37.5 ± 3.4	37.3 ± 4.4	38.4 ± 2.9		
$\%VO_{2norm}$ (%)	CAF	64.9 ± 10.8	62.9 ± 10.8	67.6 ± 8.7	67.3 ± 16.3	66.1 ± 8.0	63.1 ± 7.8*	0.048	0.011
	PLA	58.5 ± 11.6	56.8 ± 10.6	61.1 ± 9.4	59.5 ± 12.3	60.8 ± 8.1	58.7 ± 6.5		
VO_2/HR (ml/beat)	CAF	16.1 ± 2.9	14.8 ± 2.1	15.5 ± 1.5	15.4 ± 3.5	14.4 ± 2.3	15.1 ± 1.7	0.477	0.267
	PLA	15.1 ± 2.9	14.7 ± 3.9	15.2 ± 2.1	14.3 ± 2.3	14.8 ± 2.2	14.7 ± 1.5		

Values are presented as mean ± SD. CAF, caffeine; PLA, placebo; RPE, rating of perceived exertion; VO_2/kg, relative oxygen uptake; VO_2/HR, oxygen pulse; $\%VO_{2norm}$, percentage of predicted normal oxygen uptake; VE/VO_2, ventilatory equivalent for oxygen; VE/VCO_2, ventilatory equivalent for carbon dioxide; *p*-values represent the main effect of condition.

Supplementary Material Table S4

Condition × time interaction effects on physiological responses during RPE-regulated cycling.

Variable	RPE	F (df1, df2)	Interaction p-value	Partial η^2
VO_2 (L/min)	4	F (2.96, 32.51) = 0.04	0.986	0.004
	7	F (3.06, 33.65) = 0.39	0.764	0.034
VCO_2 (L/min)	4	F (1.95, 21.45) = 4.30	0.068	0.241
	7	F (2.84, 31.25) = 0.27	0.835	0.024
VE (L/min)	4	F (2.94, 32.37) = 0.27	0.842	0.023
	7	F (3.55, 39.09) = 0.24	0.893	0.022
VT (L)	4	F (1.86, 20.47) = 0.72	0.490	0.061
	7	F (2.71, 29.79) = 1.15	0.344	0.094
BF (breaths/min)	4	F (2.24, 24.69) = 1.06	0.367	0.088
	7	F (2.91, 32.06) = 0.38	0.746	0.033
RER	4	F (2.08, 22.92) = 1.34	0.283	0.108
	7	F (2.75, 30.21) = 2.07	0.130	0.158
VE/VO_2	4	F (3.31, 36.45) = 2.59	0.059	0.251
	7	F (3.28, 36.12) = 0.41	0.762	0.031
VE/VCO_2	4	F (3.19, 35.08) = 3.73	0.066	0.261
	7	F (2.32, 25.56) = 0.15	0.890	0.013
$PETO_2$ (mmHg)	4	F (2.22, 24.43) = 3.84	0.052	0.219
	7	F (2.82, 31.05) = 0.67	0.567	0.058
$PETCO_2$ (mmHg)	4	F (1.95, 21.45) = 4.30	0.028	0.281
	7	F (2.84, 31.25) = 0.27	0.835	0.024
$\%VO_{2norm}$ (%)	4	F (2.94, 32.32) = 0.05	0.983	0.005
	7	F (3.23, 35.54) = 0.07	0.980	0.006
$\%VO_{2peak}$ (%)	4	F (2.94, 32.32) = 0.05	0.983	0.005
	7	F (3.23, 35.54) = 0.07	0.980	0.006
HR	4	F (2.13, 23.46) = 0.75	0.493	0.064
	7	F (1.53, 16.80) = 0.40	0.624	0.035
VO_2/HR (mL/beat)	4	F (2.71, 29.82) = 0.02	0.995	0.002
	7	F (2.84, 31.22) = 0.59	0.615	0.051

Condition × time interactions were assessed using separate two-way repeated-measures ANOVAs at RPE 4 and RPE 7, with condition (caffeine vs. placebo) and time (2, 4, 6, 8, and 10 min) as within-subject factors. F statistics, degrees of freedom, p-values, and partial eta squared (ηp^2) are presented. Greenhouse–Geisser corrections were applied when the assumption of sphericity was violated. Statistical significance was set at $p < 0.05$.
